# Association of meleanocortin 4 receptor gene polymorphism (MC4R:C.92C>T) with feline diabetes

**DOI:** 10.5455/javar.2024.k825

**Published:** 2024-09-30

**Authors:** Kristina Morkūnienė, Justina Dambrauskaitė, Renata Bižienė, Ramutė Mišeikienė, Nijolė Pečiulaitienė, Laimutis Kučinskas, Ugnė Dudonytė

**Affiliations:** Institute of Biology Systems and Genetic Research, Lithuanian University of Health Sciences, Kaunas, Lithuania

**Keywords:** *Diabetes mellitus*, feline, inheritance, *MC4R* gene

## Abstract

**Objective:**

*Diabetes mellitus* (DM) is a prevalent endocrine condition found in cats. Recent research has identified a connection between a higher risk of diabetes in cats and genetic factors. This genetic susceptibility to type 2 diabetes is associated with genes that control insulin secretion and function, as well as those that impact factors like obesity. The purpose of the study was to determine the prevalence of the melanocortin 4 receptor (MC4R) gene missense mutation among cats and to investigate the relationship between body condition score (BCS) and the occurrence of diabetes in felines.

**Materials and Methods:**

Genotype analysis was done for 45 samples. The research results were obtained with the polymerase chain reaction-restriction fragment length polymorphism (PCR-RFLP) method. The 1,200-bp PCR product was cut with *BstOI* restriction enzyme.

**Results:**

Upon analyzing the data, it was found that 37.8% (*n* = 17) of the subjects carried the *MC4R* gene mutation. Across the entire population of cats studied, the C allele associated with the mutation was most prevalent in the group of cats with DM (frequency of 0.3) and least common among the clinically healthy cats (frequency of 0.2).

**Conclusion:**

After analysis of the study results, a statistically significant difference was observed between cats with DM and clinically healthy cats in the comparison of their BCS (*p* < 0.05). The association of the *MC4R* gene polymorphism with overweight and the development of diabetes has been identified.

## Introduction

Feline *Diabetes mellitus* (DM) stands as one of the most prevalent endocrine system disorders in cats [1]. It manifests as a syndrome characterized by hyperglycemia, which can arise from one or both causative factors—defects in insulin secretion and defects in insulin sensitivity within target tissues [2]. Insulin is exclusively produced by the pancreatic Langerhans’ Islets’ ß cells, and insulin deficiency arises when ß cells are destroyed or their function is compromised [3]. When pancreatic cells fail to produce an adequate amount of insulin or function improperly, blood sugar levels remain elevated, potentially leading to various symptoms, such as increased thirst, frequent urination, weight loss, lethargy, and others [4,5].

Type 2 DM is a complex condition where a combination of environmental and genetic factors plays a significant role in disease progression. Susceptibility to type 2 DM results from the interplay of environmental and lifestyle factors, most commonly linked to obesity, sedentary habits, corticosteroid usage, and secondary insulin resistance [6]. However, it is also acknowledged that type 2 DM has a substantial genetic and epigenetic basis [7,8].

Maintaining genetic diversity necessitates effective implementation of conservation priorities and sustainable management strategies, which should rely on comprehensive data on population structures, encompassing genetic diversity within and between breeds [9]. Genetic diversity is crucial for genetic advancement, population preservation, evolution, and adaptation to changing environmental conditions [10]. Additionally, identifying gene polymorphisms is vital in breeding [11], as it helps determine the genotypes of animals and plants and their relationships with health, reproductive performance, and economic traits [12].

To elucidate the causes behind the development of type 2 DM, several genetic studies have been conducted, including genome-wide association studies, which have identified over 50 genes associated with this condition [13,14]. Most of these genes are tied to pancreatic ß cell biology, though the mechanisms by which they relate to impaired functions and type 2 DM are still fundamentally unclear [15]. During research, it was determined that a mutation in the melanocortin 4 receptor (*MC4R)* gene is linked to obesity and an increased risk of DM in cats [4]. This implies that the genetic attributes of cats may influence the development of the disease.

Additionally, human genome association studies have identified genes linked to obesity and DM, such as the MC4R gene, which is crucial in regulating energy balance and appetite [16,17]. The MC4R gene encodes a G protein-coupled transmembrane receptor that is associated with an increased risk of developing obesity and diabetes [18]. This receptor is primarily found in the hypothalamus and plays a significant role in controlling energy balance and appetite. Under conditions of positive energy balance, MC4R is activated by alpha-melanocyte-stimulating hormone, resulting in a feeling of satiety. Conversely, during fasting, MC4R activity is suppressed by its antagonist, agouti-related protein, which triggers hunger sensations [19].

Mutations in this gene represent the most common single genetic cause of human obesity, accounting for up to 6% of cases [20]. The correlation between *MC4R *mutations and human type 2 diabetes has been demonstrated in several studies [18,21,22]. Considering the impact of this gene’s polymorphism on the likelihood of obesity and the manifestation of diabetes in humans, and recognizing obesity as a significant risk factor for feline DM as well, *MC4R *is regarded as a plausible candidate gene in the search for genetic factors that predispose cats to diabetes [16].

In 2014, the association between polymorphism of the MC4R gene and overweight domestic shorthair cats with diabetes was investigated. Among healthy overweight and healthy lean cats, the frequency of MC4R:c.92C>T alleles or genotypes did not exhibit significant differences. However, within the group of overweight cats with diabetes, 55% of animals were homozygous for the MC4R:c.92C allele, compared to 33% of lean cats with diabetes and 30% of non-diabetic cats. The differences between overweight cats with diabetes and non-diabetic cats were statistically significant. The work aimed to investigate the polymorphism (MC4R:c.92C>T) by polymerase chain reaction-restriction fragment length polymorphism (PCR-RFIP) method in healthy and diabetic cats bred in Lithuania and to analyze the relationship between feline DM, body condition, gender, and heredity.

## M**aterials** **and** M**ethods**

Ethical approval was obtained for the study (Ethics approval No. BEC-VM-01, Appendix No. 1). Biological samples (buccal epithelial cells) were collected from cats. A total of 94 (45 females, 49 males, various ages) samples were collected and analyzed for the study. However, results were successfully obtained from only 45 (22 females and 23 males) samples; among them, 30 samples were from clinically healthy cats, and 15 were from cats diagnosed with DM. Although the determination of DNA purity and concentration showed that a sufficient amount of DNA was extracted from all the tested samples, a significant portion of fragments did not separate during electrophoresis or appeared very faint, making it impossible to identify all cat genotypes. The exact reason for the failure to obtain results from a substantial number of samples is not clear. However, it is suspected that the obtained DNA concentration might have been inadequate, or the PCR primers failed to anneal successfully. Therefore, for future iterations of this study, it is recommended to collect blood samples with higher DNA concentrations instead of buccal epithelial cells.

The samples were collected from various breeds of cats, including British Shorthairs, Persians, Russian Blues, Cornish Rexes, and others. Information about the cat’s breed, age, gender, weight, neutering status, and body condition score (BCS) was gathered from the owners. Owners of cats diagnosed with diabetes were also asked about the timing of their cat’s diabetes diagnosis and the medications currently being used.

**Figure 1.**
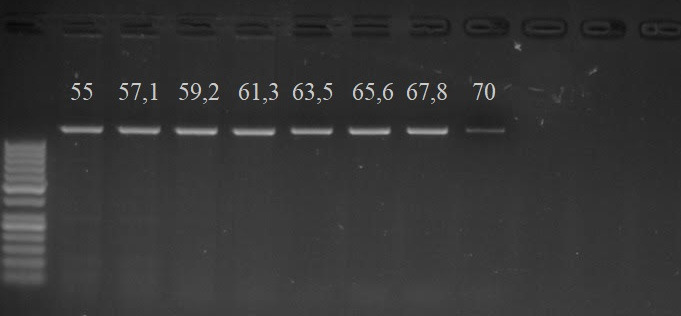
Optimization of the study using the gradient PCR method, indicating the applied temperatures (°C) at the top, PCR product size 1,200-bp.

The DNA extraction from buccal epithelial cells was carried out using the method outlined by Aidar and Line [23]. The *MC4R *gene polymorphism study was performed by the PCR-RFLP method. This study enables the identification of a mutated allele on the D3 chromosome of the *MC4R* gene, which arises due to a missense mutation where leucine changes to proline (CTT/CCT) at the 31st position of the first exon, associated with the development of DM in cats. Specific primers encompassing the 5’ untranslated region (UTR; forward: 5’‐CTC AGA ACT TTC GGG CAG AC‐3’) and the 3’ UTR (reverse: 5’‐ACC CAT GCC TTA CAC AGA GG‐3’) were designed based on the existing sequence of the feline *MC4R* gene (Ensembl database; Genome assembly Felis_catus-6.2). At the beginning of the study, an investigation of the feline* MC4R* gene was conducted using the gradient PCR method to determine the optimal primer annealing temperature (Fig. 1).

The restriction enzyme *BstOI* recognizes the cleavage site (C**C**(A/T)GG) when the MC4R:c.92C allele is present, while in the case of the MC4R:c.92T allele, it lacks an additional cleavage site. A 10 μl PCR product was digested with a 10 μl restriction mixture (7.5 μl deionized water, 2 μl buffer, 0.5 μl* BstOI*). Subsequently, the PCR products with the restriction mixture were incubated in a thermostat for 3–16 h at a temperature of 37°C (Table 1). The digested PCR products were fractionated by electrophoresis on 2% agarose gel (1 × TAE) stained with 10 μl ethidium bromide at 110 V for 45 min. Subsequently, it was analyzed under UV light (wavelength 300 nm) using the “MiniBisPro” video documentation system (Herolab, Germany).

All procedures adhered to the requirements of the National and European Union legislation: the Law of the Republic of Lithuania on the Protection, Care, and Use of Animals No. VIII-500/1997; and the European Convention for the Protection of Vertebrate Animals Used for Experimental and Other Scientific Purposes. Ethical approval was obtained for the study (Ethics approval No. BEC-VM-01, Appendix No. 1).

### Statistical analysis

The analyses were conducted using the “IBM SPSS Statistics” software. To assess the influence of breed, gender, neutering, age, and onset on susceptibility, “descriptive statistics” and “Crosstabs” functions were employed, and reliability was computed using the “*chi*-square” test. When calculating the frequencies of age groups and breeds, the “descriptive statistics” “frequency” function was used, and the average age was calculated utilizing the “Compute variable” “mean” function. Additionally, the dependency of genotype on breed, gender, and onset was evaluated using the “Descriptive statistics” “Crosstabs” function, and reliability was determined through the “*chi*-square” test. Results were considered significant when *p *< 0.05.

## Results

Results were obtained for a total of 45 cats under investigation. The animals were divided into two groups: the diabetic group and the control group. The diabetic group consisted of 15 cats with DM, while the control group included 30 clinically healthy cats. Both groups comprised representatives of various cat breeds, although the majority were mixed-breeds (57.8%).

**Figure 2.**
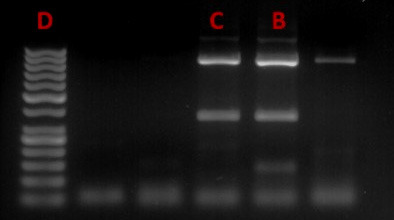
Genotype analysis: B – T/C genotype; C – T/T genotype; D – 50-bp molecular weight marker (Thermo Fisher Scientific).

**Figure 3.**
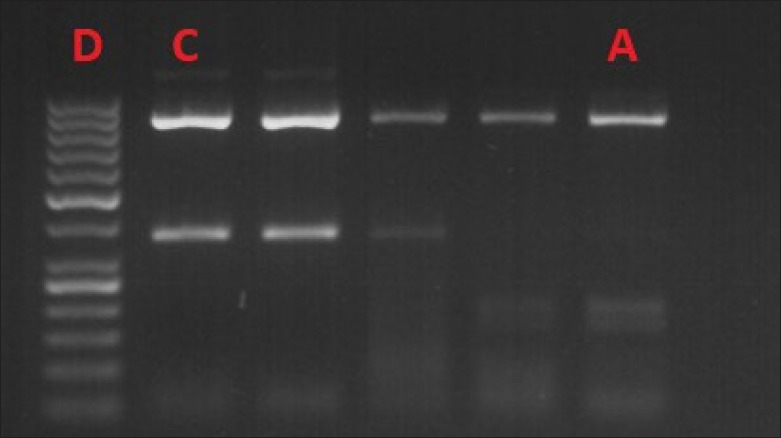
Genotype analysis: A – C/C genotype; C – T/T genotype; D – 50-bp molecular weight marker (Thermo Fisher Scientific).

*MC4R* gene c.92T>C DNA fragment sizes after digestion with the restriction enzyme were: homozygous genotype for the normal allele (T/T): 885, 315-bp; heterozygous genotype (T/C): 885, 315; 165, and 150-bp; homozygous genotype for the mutated allele (C/C): 885, 165, and 150-bp (Figs. 2 and 3).

**Figure 4.**
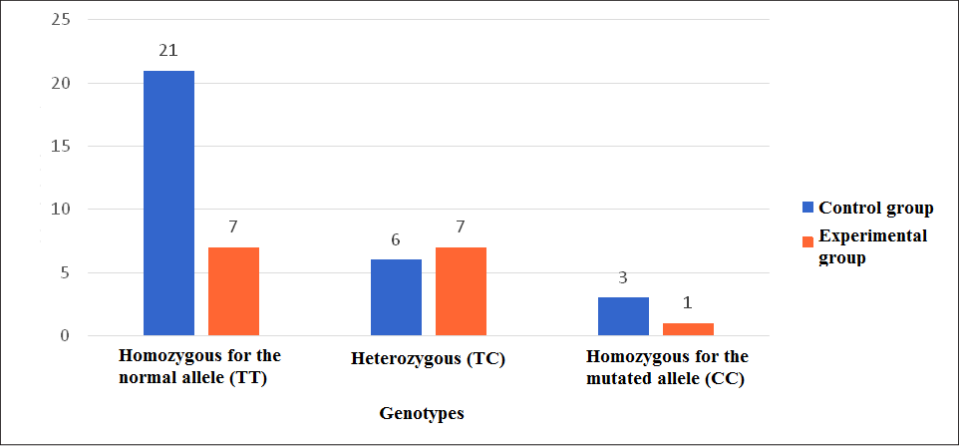
Genotypes distribution among diabetic and control groups.

Upon analyzing the obtained results, the homozygous genotype for the normal allele (T/T) was identified in 7 (15.6%) cats from the diabetic group and 21 (46.6%) cats from the control group. The heterozygous genotype (T/C) was determined in 7 (15.6%) cats from the diabetic group and 6 (13.3%) cats from the control group. The homozygous genotype with both mutated alleles was found in 1 (2.2%) cat from the diabetic group and 3 (6.7%) cats from the control group (Fig. 4).

The frequencies of genotypes were examined separately for the diabetic, control, and all studied animal groups. From the results, it can be observed that among all studied cats (0.62) and in the control group (0.7), the homozygous genotype for the unchanged allele (T/T) was the most frequent. In the diabetic group, the frequency of this genotype (0.47) was the same as that of the heterozygous genotype (T/C). When comparing the frequencies of the homozygous genotype for the mutated allele (C/C), the highest occurrence was in the control group of cats (0.1), while the lowest was in the diabetic group of cats (0.06) (Table 2).

The allele frequencies were calculated for the diabetic, control groups, and among all studied cats. The most commonly recurring allele in all groups was T, with the lowest occurrence observed in the diabetic group (0.7). Among all groups of individuals, the frequency of the C allele was highest in the diabetic group (0.3), while it was lowest in the control group (0.2) (Table 3).

Based on the obtained research data, the distribution of genotypes according to gender was assessed, and the influence of gender on DM susceptibility was determined, along with the calculation of result reliability. When comparing all cats, female cats affected by DM comprised 17.8%, while clinically healthy females were 31.1%. Male cats affected by DM accounted for 15.6%, whereas clinically healthy males accounted for 35.5%. Therefore, although the results were statistically insignificant (*p* = 0.673), a higher proportion of females were affected by diabetes.

**Table 1.** PCR reaction conditions, size of PCR product, and restriction enzyme.

Genetic defect	PCR profile			PCR product size	Restriction enzyme
DM	95°C	10 min		1,200-bp	*BstOI*
	94°C	40 sec	30 cycles		
	65°C	30 sec			
	72°C	2 min			
	72°C	10 min			

**Table 2.** Genotypes frequency table of *MC4R* gene in studied cats population.

Genotypes	C/C	T/C	T/T
Frequencies of all tested cat genotypes	0.09	0.29	0.62
Frequencies of diabetic group cat genotypes	0.06	0.47	0.47
Frequencies of control group cat genotypes	0.1	0.2	0.7

Evaluating the genotype distribution by gender, among females, 63.6% had the homozygous genotype for the unchanged allele (T/T), 31.8% had the heterozygous genotype (T/C), and only one female had the homozygous genotype for the mutated allele (C/C). Among males, about 61% had the homozygous genotype for the unchanged allele (T/T), around 26% had the heterozygous genotype (T/C), and 13% had the homozygous genotype for the mutated allele (C/C). While statistically significant results were not obtained when comparing the gender’s influence on the genotype (*p* = 0.59), a higher percentage of males had the homozygous genotype for the mutated allele (C/C), and the homozygous genotype for the unchanged allele (T/T) was present in an equal number of males and females.

**Table 3.** Allele frequency table of *MC4R* gene in studied cats population.

Allele	All tested cats	Diabetic group	Control group
C	0.23	0.3	0.2
T	0.77	0.7	0.8

**Table 4.** BCS distribution.

BCS	3	4	5	6	7	8
Diabetic	0	3	3	4	4	1
Clinically healthy	2	22	4	1	1	0

**Table 5.** Genotype distribution between lean and obese cats with CD and clinically healthy cats

Genotypes	CC	CT/TT	Total
Lean with CD	1	5	6
Obese with CD	0	9	9
Clinically healthy	3	27	30

An assessment of the influence of body condition was performed. Body condition was evaluated based on the BCS, ranging from 1 to 9 points [20]. During the study, cats with a BCS of 6 or higher were classified as overweight or obese, while those with a score of 5 or lower were classified as lean. Among cats affected by DM, the most frequently recurring BCS were 6 and 7, while clinically healthy cats showed scores of 4 and 5 (Table 4). The average score among cats with DM was 5.8, while in clinically healthy ones, it was 4.2. When evaluating the impact of body condition on DM susceptibility, statistically significant results were obtained (*p* < 0.05). Additionally, the genotype distribution was calculated considering the body condition; however, the obtained results were statistically insignificant (*p* = 0.802).

For the comparison of genotypes among lean and obese cats affected by DM and clinically healthy individuals, Table 5 was compiled. The homozygous genotype for the mutated allele (C/C) was present in 2.2% of lean cats with DM and 6.7% of clinically healthy cats. The heterozygous (C/T) and homozygous genotypes for the unchanged allele (T/T) were observed in 11.1% of lean cats with DM, 20% of obese cats with DM, and 60% of clinically healthy cats. However, the obtained data were statistically insignificant (*p* = 0.115).

## Discussion

The prevalence of the MC4R c.92C>T mutation was studied in a selected population of felines. All cats were divided into two groups: the diabetic group (cats with DM) and the control group (healthy cats).

The heterozygous (T/C) genotype was identified in 15.6% of cats in the diabetic group and 13.3% in the control group. The homozygous C/C genotype with both mutated alleles was found in 2.2% of cats in the diabetic group and 6.7% in the control group. Consequently, when considering genotypes with at least one mutated allele, they were more common in the clinically healthy cat group than the affected cat group.

Upon genotype frequency calculation, it was observed that the T/T genotype without mutation was most prevalent in the control group of cats (0.7). Assessing the frequency of the heterozygous T/C genotype, it was found that in the diabetic group (0.47), this genotype’s frequency was over two times greater than in the control group (0.2). Furthermore, evaluating allele frequencies across all animal groups, the C allele frequency was highest in the study group (0.3) and lowest in the control group (0.2).

Comparing the influence of gender on susceptibility to feline diabetes, affected female cats constituted 17.8%, while males accounted for 15.6%. Thus, despite a slight difference, females comprised a larger portion of affected cats. Nonetheless, it is important to note that in evaluating genotype distribution by gender, a higher proportion of males possessed the homozygous mutated allele genotype (C/C). When comparing the obtained data with the results of an epidemiological study conducted in England [24], males were 1.6 times more likely to develop DM than females. However, considering other risk factors such as weight, gender did not have a significant impact on a patient’s risk of developing DM [25]. In studies conducted in Sweden, males were twice as likely to develop diabetes compared to females, and these results were statistically significant [26].

In the conducted study of cats with DM, the most frequent body composition indices were 6 and 7 for affected cats and 4 and 5 for clinically healthy cats. The average body composition index for cats with DM was 5.8, and for clinically healthy cats, it was 4.2. Evaluating the influence of body composition on susceptibility revealed statistically significant results (*p* < 0.05). Therefore, overweight cats had a higher likelihood of developing diabetes. This hypothesis is supported by analyses of data conducted by other authors [27,28].

Additionally, the study results were compared with research conducted by scientists in 2014 [12], and a table describing the distribution of genotypes among cats afflicted with lean or obese DM and clinically healthy cats was created. According to Forcada et al. [14,15], the C/C genotype was most common among overweight cats with DM (55%), while this genotype was characteristic of about a third of lean cats with DM and clinically healthy cats. Differences between obese cats with DM and clinically healthy cats were statistically significant (*p* < 0.01). In the selected cat population genotype study, different results were obtained: 2.2% of lean cats with DM had the C/C genotype, and 6.7% of clinically healthy cats had it. Meanwhile, the C/T and T/T genotypes were present in 11.1% of lean cats with DM, 20% of obese cats with DM, and 60% of clinically healthy cats. However, the sample size of the study population was considerably smaller, resulting in statistically insignificant results (*p* = 0.115).

In the study of the selected cat population, the calculated average age of DM onset was 9 years. In other studies, the average age of diagnosis ranged from 10.7 years with a standard deviation of 3.1 years [25] to 10.9 years with a standard deviation of 3.1 years [26]. A study in 2016 indicated that the risk of DM increased with patient age, starting at 6 years [24]. Based on the collected data, 50% of the adult cat population in the selected sample suffered from DM, while the proportion of DM-affected senior cats reached 90%, and only 10% were clinically healthy. Assessing the impact of age groups on susceptibility yielded statistically significant results (*p* < 0.01). This conclusion aligns with findings from other studies, which suggest a higher risk of DM in middle-aged and geriatric cats [24–26].

## C**onclusion**

Cats most commonly manifest type 2 diabetes, typically resulting from pancreatic ß-cell insufficiency alongside cellular insulin resistance due to obesity. According to the latest scientific research, a connection has been established between an increased risk of feline diabetes and heredity. The genetic predisposition to type 2 DM is associated with genes controlling insulin secretion and function, as well as genes influencing factors like susceptibility to obesity. The association of the MC4R gene polymorphism with overweight and the development of diabetes has been identified. In the selected cat population, at least one mutated allele of the MC4R gene was present in the genotype of 37.8% of animals. 28.9% of felines had the heterozygous genotype (T/C), and 8.9% had the homozygous genotype with both mutated alleles (C/C). After data analysis, it was determined that the frequency of the mutated C allele was highest in the group of cats affected with diabetes and lowest in clinically healthy cats.

## List of Abbreviations

DM, *Diabetes Mellitus*; *MC4R*, Melanocortin 4 receptor (MC4R) gene; PCR-RFLP, Polymerase chain reaction-restriction fragment length polymorphism.
